# Prevalence of self-reported myocardial infarction in Sami and non-Sami populations: the SAMINOR study

**DOI:** 10.3402/ijch.v74.24424

**Published:** 2015-01-12

**Authors:** Bent-Martin Eliassen, Sidsel Graff-Iversen, Tonje Braaten, Marita Melhus, Ann R. Broderstad

**Affiliations:** 1 Centre for Sami Health Research, Department of Community Medicine, Faculty of Health Sciences, UiT The Arctic University of Norway, Tromsø, Norway; 2 Norwegian Institute of Public Health, Nydalen, Oslo, Norway; 3 Department of Community Medicine, Faculty of Health Sciences, UiT The Arctic University of Norway, Tromsø, Norway; 4 Department of Medicine, University Hospital of Northern Norway, Harstad, Norway

**Keywords:** cardiovascular disease, indigenous, Norway

## Abstract

**Objective:**

Measure the prevalence of self-reported myocardial infarction (SMI) in Sami and non-Sami populations in rural areas of Norway, and explore whether possible ethnic differences could be explained by established cardiovascular risk factors.

**Design:**

Cross-sectional population-based study.

**Methods:**

A health survey was conducted in 2003–2004 in areas with Sami and non-Sami populations (SAMINOR). The response rate was 60.9%. Information concerning lifestyle was collected by 2 self-administrated questionnaires, and clinical examinations provided anthropometric measurements, and data on blood pressure and lipid levels.

**Results:**

The total number for the subsequent analysis was 15,206 men and women aged 36–79 years (born 1925–1968). Sex-specific analyses revealed no ethnic difference in SMI. In terms of the most important risk factors such as smoking, blood pressure, and lipid levels, no or only trivial ethnic differences were found in both women and men.

**Conclusion:**

In this study, we found no difference in SMI between Sami and non-Sami in rural areas in Norway. The similar risk profile is the most plausible explanation; similar living conditions and close interaction between the ethnic groups may explain this.

The Sami are an indigenous people whose settlement area (Sápmi) covers the northern parts of Norway, Sweden and Finland, and Russia's Kola Peninsula. The traditional Sami settlement area in Norway stretches from Finnmark in the north to Engerdal in Hedmark County in the south.

In the latter half of the 20th century, Sami and non-Sami populations in Norway underwent lifestyle changes resulting in more sedentary lifestyles and dietary changes (1,2). The knowledge concerning the burden of myocardial infarction among the Sami is limited, and we do not know whether the prevalence of this disease differs between Sami and non-Sami populations in Norway. Some studies have, however, been carried out; for example, the first Finnmark study was initiated in 1974–1975, wherein Sami/Finnish men aged 35–49 years had on average a 40% higher cardiovascular risk score compared with Norwegians (3). The score was based on sex, serum total cholesterol, systolic blood pressure and current cigarette smoking (ibid.). Despite this risk profile, the prevalence of self-reported myocardial infarction (SMI) was considerable lower in the Sami compared with the Norwegian population (ibid.). Since then, prevalence and follow-up data have shown no or only minor differences in risk factors and risk of ischaemic heart disease (IHD) (2,4–10). In terms of mortality from cardiovascular disease, conflicting results have been presented (11,12). Various definitions of Sami ethnicity have been used in these studies.

The primary objective of this study was to measure the prevalence of SMI in Sami and non-Sami populations, and to explore whether possible differences could be explained by established cardiovascular risk factors.

## Methods

As part of the Norwegian Institute of Public Health's (NIPH) health survey in Finnmark, the Centre for Sami Health Research conducted in 2003–2004 a population-based survey in areas with Sami and non-Sami populations (SAMINOR) (13), also including areas outside Finnmark. The age cohorts included in SAMINOR were 30 and 36–79 year-olds. All eligible residents in the selected municipalities registered in the National Population Register were invited regardless of ethnic background (N=27,987). Of these, 16,865 (60.3%) participated and gave consent to medical research. The response rate in the age group 36–79 (n=16,538) was 60.9%. The population was almost exclusively rural. The Regional Committee for Medical Research Ethics approved the study and the participants gave written informed consent.

### Questionnaires

Information on ethnicity and lifestyle was collected by using 2 questionnaires, that is, the initial and main questionnaires (13). Ethnicity was measured by the following: *What language(s) do/did you, your parents and your grandparents use at home?* The response categories were “Norwegian,” “Sami,” “Kven” or “other.” Kvens are descendants of Finnish settlers who immigrated to northern Norway in the 1700s and 1800s (14). Providing the same response options, we also asked: *What is your, your father's and your mother's ethnic background?* The respondents also reported whether they considered themselves to be Norwegian, Sami, Kven or other (self-perceived ethnicity). On all these questions multiple answers were allowed. These variables are described in detail by Lund et al. (13). Based on these variables we generated 2 ethnic categories: Sami and non-Sami. The Sami category included respondents reporting at least one Sami identity mark (Sami language spoken by the respondent or at least one parent or grandparent, or Sami ethnic background or self-perceived Sami ethnicity). Those not reporting a Sami identity mark were included in the non-Sami group.

Myocardial infarction was measured by the following question: *Do you have, or have you ever had: A heart attack?* (Yes/No). Missing values (n=640) were considered negative responses; for such variables, this is a conventional ad-hoc imputation method (15).

The following question measured family history of myocardial infarction: *Tick off relatives who have, or have ever had, any of the following conditions, and report the age of when they got the illness*: “Heart attack before the age of 60 years” and “Heart attack after the age of 60 years.” The possible response categories were “Mother,” “Father,” “Sister,” “Brother,” “Children” and “No one.” The variables were combined to create a variable of total family history of myocardial infarction. Observations providing a negative response but reporting the age for when their relative(s) got the illness were given a positive response (n=9).

Data on smoking were collected by asking: *Are you currently, or were you previously, a daily smoker*? “Yes, currently,” “Yes, previously,” “Never.” Never smokers (n=37), current smokers (n=86) and observations with missing data on smoking status (n=14) reporting the number of years since they stopped smoking were coded as previous smokers.

Education was measured by asking: *How many years of schooling/education have you completed (including all the years you attended school or have been studying)*?

Use of cholesterol-lowering drugs during the last 4 weeks was registered by the following item: *State the name of the medicine and your reason for taking/having taken them (disease, symptoms): Tick one box for each line*. Relevant ATC reference codes were used.

### Medical examination

A trained group of experienced fieldworkers from the NIPH performed the medical examinations, which took place in 2 buses traveling between the municipalities. Invitations with the time and date for the medical examination were sent in the mail together with the questionnaires. Waist circumference was measured at the umbilicus to the nearest centimetre at the end of exhalation with the individual standing and breathing normally. The methods used and procedures followed for the measurement of blood pressure, total cholesterol, high-density lipoprotein (HDL) cholesterol and glucose are described in detail elsewhere (9). All blood samples in the SAMINOR study were non-fasting. We used self-reported diabetes and information about anti-diabetic medication to define diabetes. Additionally, a non-fasting blood glucose level ≥11.1 was defined as having diabetes (16).

### Statistical analysis

Means in Tables I and II were tested by 2-sample t-tests; rates were tested by Pearson's chi-square tests or 2-sample Wilcoxon rank-sum (Mann–Whitney) tests. The distributions of HDL cholesterol and glucose were skewed and thus log-transformed; the displayed means are geometric. The direct method was used to age-standardize the prevalence of total SMI (Table III) to the European standard population of 1976 (17). Logistic regression was then used to evaluate the effects of the explanatory variables on the relationship between ethnicity and SMI (Table IV). Systolic blood pressure, HDL cholesterol and total cholesterol were included in the regression models as categorical variables, as displayed in Tables I and II. Appropriate goodness-of-fit tests were performed to optimize the regression models. Alcohol consumption, leisure-time physical activity, and intensity and duration of smoking (pack-years in current smokers and time since smoking cessation in previous smokers (18) were also included in the regression models to assess any additional confounding effects. We also performed the regression analysis with and without the ad-hoc imputation to assess if the imputations of negative SMI cases influenced estimates and results in Table IV.

**Table I:** Characteristics of the female study group by ethnicity. Values are means^a^ or percentages^b^ (95% CI); p-values for differences between ethnic groups (SAMINOR study 2003–2004, n=15,206^c^)

	Non-Sami	Sami	
			
Variables	n = 5,273	n = 2,611	p
Age (years)	54.5 (54.2–54.8)	54.2 (53.7–54.6)	0.21
Systolic blood pressure (mmHg)	130.4 (129.8–131.0)	129.7 (128.8–130.5)	0.13
Minimum–119	35.7 (34.4–37.0)	36.5 (34.7–38.3)	0.17
120–129	17.6 (16.6–18.7)	19.5 (18.0–21.0)	
130–139	16.9 (15.9–17.9)	15.8 (14.4–17.2)	
140–149	12.2 (11.3–13.1)	11.3 (10.1–12.5)	
150–159	8.4 (7.7–9.2)	7.6 (6.6–8.6)	
160 +	9.2 (8.4–10.0)	9.5 (8.4–10.7)	
Total cholesterol (mmol/l)	5.99 (5.96–6.02)	5.98 (5.93–6.02)	0.62
Minimum–4.09	3.2 (2.7–3.7)	3.5 (2.9–4.3)	0.87
4.10–5.19	23.3 (22.1–24.4)	22.3 (20.7–23.9)	
5.20–6.29	36.7 (35.4–38.0)	36.3 (34.5–38.2)	
6.30–7.29	22.9 (21.7–24.0)	25.7 (24.0–27.4)	
7.3 +	14.0 (13.1–15.0)	12.2 (11.0–13.5)	
HDL cholesterol (mmol/l)^d^	1.44 (1.43–1.45)	1.40 (1.39–1.42)	<0.001
1.61 +	34.2 (33.0–35.5)	29.4 (27.7–31.2)	<0.001
1.30–1.60	33.1 (31.8–34.4)	33.5 (31.7–35.3)	
1.20–1.29	10.3 (9.6–11.2)	10.0 (8.9–11.2)	
0.90–1.19	19.2 (18.1–20.3)	23.3 (21.8–25.0)	
Minimum–0.89	3.2 (2.7–3.7)	3.8 (3.1–4.6)	
Type 2 diabetes (yes)	4.3 (3.8–4.8)	5.0 (4.3–5.9)	0.12
Glucose (mmol/l)^d^	5.44 (5.41–5.47)	5.50 (5.46–5.55)	<0.05
Statin use (yes)	11.3 (10.4–12.2)	11.9 (10.7–13.2)	0.43
Family history of MI (yes)	47.1 (45.7–48.4)	51.2 (49.3–53.2)	<0.001
Education (≥13 years) (yes)	34.2 (32.9–35.5)	33.5 (31.6–35.4)	0.56
Smoking			0.18
Never	37.6 (36.3–38.9)	36.4 (34.5–38.2)	
Previous	31.3 (30.1–32.6)	31.0 (29.3–32.8)	
Current	31.1 (29.9–32.4)	32.6 (30.8–34.4)	

a Tested by 2-sample t-test with equal variances.

b Tested by Pearson's chi-square test or 2-sample Wilcoxon rank-sum (Mann–Whitney) test.

c Some estimates are based on a smaller n due to missing values.

d Geometric mean.

**Table II:** Characteristics of the male study group by ethnicity. Values are means^a^ or percentages^b^ (95% CI), p-values for differences between ethnic groups (SAMINOR study 2003–2004, n=15,206^c^)

	Non-Sami	Sami	
			
Variables	n=4,746	n=2,576	p
Age (years)	54.8 (54.5–55.1)	55.0 (54.6–55.4)	0.50
Systolic blood pressure (mmHg)	134.2 (133.7–134.7)	134.9 (134.2–135.7)	0.13
Minimum–119	21.5 (20.3–22.7)	22.1 (20.5–23.7)	0.56
120–129	21.3 (20.1–22.5)	21.0 (19.4–22.6)	
130–139	23.8 (22.6–25.0)	22.2 (20.6–23.9)	
140–149	15.6 (14.6–16.6)	14.9 (13.6–16.3)	
150–159	8.8 (8.0–9.6)	9.5 (8.4–10.7)	
160 +	9.2 (8.4–10.0)	10.4 (9.3–11.6)	
Total cholesterol (mmol/l)	5.89 (5.86–5.92)	5.98 (5.93–6.02)	<0.01
Minimum–4.09	4.2 (3.6–4.8)	4.7 (4.0–5.6)	<0.001
4.10–5.19	22.8 (21.6–24.0)	21.8 (20.3–23.5)	
5.20–6.29	39.7 (38.3–41.1)	34.5 (32.7–36.4)	
6.30–7.29	23.4 (22.3–24.7)	26.3 (24.6–28.0)	
7.3 +	9.9 (9.1–10.8)	12.6 (11.4–14.0)	
HDL cholesterol (mmol/l)^d^	1.21 (1.20–1.22)	1.23 (1.21–1.24	0.12
1.61 +	13.8 (12.9–14.8)	15.0 (13.7–16.5)	0.32
1.30–1.60	24.6 (23.4–25.9)	24.9 (23.3–26.6)	
1.20–1.29	13.0 (12.1–14.0)	11.3 (10.1–12.5)	
0.90–1.19	37.6 (36.3–39.0)	38.6 (36.8–40.5)	
Minimum–0.89	11.0 (10.1–11.9)	10.1 (9.0–11.4)	
Type 2 diabetes (yes)	4.5 (4.0–5.2)	5.3 (4.5–6.2)	0.14
Glucose (mmol/l)^d^	5.61 (5.57–5.64)	5.63 (5.58–5.68)	0.50
Statin use (yes)	14.6 (13.6–15.6)	14.9 (13.6–16.3)	0.71
Family history of MI (yes)	45.8 (44.4–47.2)	49.4 (47.5–51.4)	<0.01
Education (≥13 years) (yes)	31.4 (30.0–32.7)	26.6 (24.9–28.4)	<0.001
Smoking			<0.05
Never	28.4 (27.1–29.7)	25.5 (23.9–27.3)	
Previous	41.5 (40.1–42.9)	42.5 (40.6–44.4)	
Current	30.2 (28.9–31.5)	32.0 (30.2–33.8)	

a Tested by 2-sample t-test with equal variances.

b Tested by Pearson's chi-square test or 2-sample Wilcoxon rank-sum (Mann–Whitney) test.

c Some estimates are based on a smaller n due to missing values.

d Geometric mean.

**Table III:** Age-specific, and total crude and age-standardized, prevalence rates of self-reported myocardial infarction by sex and ethnicity (SAMINOR study 2003–2004, n=15,206)

	Non-Sami			Sami		
		
Age	Sample	n	%	Sample	n	%
Women						
36–49	1,947	4	0.2	1,009	2	0.2
50–59	1,557	13	0.8	795	12	1.5
60–69	1,110	36	3.2	496	13	2.6
70–79	659	39	5.9	311	29	9.3
Total crude	5,273	92	1.7	2,611	56	2.1
Total age-adjusted^a^ (95% CI)	5,273	81	1.5 (1.2–1.9)	2,611	51	2.0 (1.5–2.5)
Men						
36–49	1,657	14	0.8	875	5	0.6
50–59	1,487	72	4.8	850	43	5.1
60–69	1,041	120	11.5	536	53	9.9
70–79	561	103	18.4	315	54	17.1
Total crude	4,746	309	6.5	2,576	155	6.0
Total age-adjusted^a^ (95% CI)	4,746	273	5.8 (5.2–6.4)	2,576	136	5.3 (4.5–6.1)

a Direct standardization using the European standard population as reference (18).

**Table IV:** Odds ratios (OR) and 95% confidence intervals (CIs) for the differences in self-reported myocardial infarction between non-Sami and Sami by sex (SAMINOR study 2003–2004, n=15,206)

	Model 1^a^			Model 2^b^		
		
	OR	p	95% CI	OR	p	95% CI
Women	n=7,884			n=7,261^c^		
Non-Sami	Ref			Ref		
Sami	1.29	0.14	0.92–1.81	1.25	0.26	0.85–1.85
Men	n=7,322			n=6,919^c^		
Non-Sami	Ref			Ref		
Sami	0.91	0.36	0.74–1.12	0.88	0.30	0.68–1.13

Controlling for:

a Age.

b Age and education, previous and current smoking, systolic blood pressure, HDL cholesterol, type 2 diabetes, family history of myocardial infarction, total cholesterol and use of statins.

c Lower sample size due to missing values.

An alternative categorization of Sami ethnicity was assessed by dichotomizing Sami ethnicity into Sami I (Sami language is used as the home language by all grandparents and parents and by the participant) and Sami II (at least one Sami identity mark, i.e. Sami language spoken by the respondent or at least one parent or grandparent, or Sami ethnic background or self-perceived Sami ethnicity).

All statistical analyses were performed using STATA version 13.0 (StataCorp, College Station, TX).

## Results

The total number for the subsequent analysis was 15,206 men and women aged 36–79 years (born 1925–1968); those aged 30 years (n=327) (low response rate and few disease events) and those who did not respond to the initial (n=207) and main (n=785) questionnaires were excluded from the analysis. Also excluded were recent immigrants (n=257), those with missing information on ethnicity (n=52) and participants who did not turn up for the medical examination (n=31). Recent immigrants were responders who were born abroad and answered “other” to the first 2 questions on ethnicity (see questionnaires).

Tables I and II display the levels of selected risk factors by ethnicity in women and men, respectively. In women, statistically significant (p<0.05) but small differences between non-Sami and Sami were found for HDL cholesterol, glucose and family history of myocardial infarction.

In men, statistically significant (p<0.05) differences were found for total cholesterol, family history of myocardial infarction, years of education and smoking. These differences were also small. In both sexes, the Sami had higher levels of hereditary myocardial infarction.

Nonetheless, in terms of the most important risk factors such as smoking, blood pressure and lipid levels, no or only trivial ethnic differences were found in both women and men.

Table III displays age-specific, and total crude and age-standardized, prevalence rates of SMI by sex and ethnic groups. Similar rates and overlapping confidence intervals were seen when comparing Sami and non-Sami women and men. In women, the total age-standardized rates for non-Sami and Sami were 1.5% (95% CI: 1.2–1.9) and 2.0% (95% CI: 1.5–2.5), respectively. The total age-standardized rates for non-Sami and Sami men were 5.8% (95% CI: 5.2–6.4) and 5.3% (95% CI: 4.5–6.1), respectively.

Odds ratios (ORs) for SMI in Sami compared with non-Sami are presented in Table IV. In both women and men, univariable and multivariable regression analyses revealed no significant ethnic difference in odds of SMI.

No confounding due to alcohol consumption, leisure-time physical activity, and intensity and duration of smoking was observed (data not shown). The ad-hoc imputation of negative cases of SMI did not affect the end result (data not shown). Furthermore, the alternative categorization of ethnicity (see methods) did not affect the results in the univariable and multivariable regression analyses (data not shown).

## Discussion

In this study, we found no difference in SMI between Sami and non-Sami in selected rural areas in Norway; the similar risk profile is the most plausible explanation. An alternative categorization of ethnicity (see methods) did not affect the end result (data not shown).

The similar risk profile and burden of SMI in Sami and non-Sami in rural areas of northern Norway are in line with those found in earlier studies (2,4–10). In Sweden, a few follow-up studies have shown little or no difference between Sami and non-Sami with regard to cardiovascular risk factors (19,20), and a somewhat higher Sami mortality rate due to IHD in women (21,22). A lower incidence rate of acute myocardial infarction has also been documented in Sami reindeer-herding women compared with Swedish women (22). Other Arctic indigenous peoples such as the Greenlandic Inuit present similar IHD morbidity rates compared to American and European populations (23).

Having used the same data as in this study, previous SAMINOR publications have already presented small ethnic differences in risk factors for CVD (2,9,10). The similar living conditions and close interaction between the ethnic groups may explain this; it is likely that the mentioned lifestyle changes have occurred in all parts of the rural population in northern Norway independent of ethnicity (9). However, in a recent publication based on the same data used in this study, Eliassen et al. (24) found an excess of self-reported angina pectoris in Sami compared with non-Sami, which was surprising given the strikingly similar risk profile. This may suggest that our grasp of IHD aetiology in the Sami population may be missing key factors; unknown genetic or environmental factors may influence the occurrence of IHD in this population.

### Limitations

Sensitivity has been found to be quite high for SMI in other populations (80–97.7%), whereas positive predictive values have been reported lower (43–87%) (25). A recent study, using the Spanish cohort of the EPIC study, found self-reported information on myocardial infarction to be a valid instrument when compared with medical records (ibid.). Previous population studies in northern Norway support agreement between SMI and medical records (26,27). This may provide some support to the validity of the self-reported data. However, at present we do not know the validity of SMI in this population and in the 2 ethnic groups; the fact that Eliassen et al. (24) found an excess burden of angina pectoris among Sami in the same sample may suggest a potential bias in this study. Body, health, and disease are cultural phenomena; there is no universal connection between a medical condition and the way it is experienced (28); and standardized questions in a self-administrated questionnaire may convey different meanings to different peoples (29). This might thus have produced differential misclassification of disease status by ethnicity.

This study had a cross-sectional design; we were unable to assess potential causal relationships. We have limited information about non-responders other than that they tended to be younger, single and male (2); potential selection bias is thus difficult to assess. We do not know the response rate by ethnicity; we are therefore unable to assess whether differences in participation have influenced the observed disease burden in the ethnic groups.

An additional limitation is that the data are approximately 10 years old; the results may therefore have reduced applicability today.

### Strengths

A relatively high overall response rate (61%) and a large population-based sample have enabled an in-depth comparative analysis of SMI in Sami and non-Sami populations. Generalizability in SAMINOR refers to whether the general population in the defined SAMINOR area systematically differs from the rural population in general in northern Norway, and whether those who participated in the study systematically differ from those not included (9). We believe that our results can be generalized to the Sami and non-Sami living in the rural areas of northern Norway. However, they may be less valid for the population in Nordland due to the low response rate in this region (9,13).

## Conclusions

In this study, we found no difference in SMI between Sami and non-Sami in rural areas in Norway. The similar risk profile is the most plausible explanation; similar living conditions and close interaction between the ethnic groups may explain this.

## References

[1] Brustad M, Parr CL, Melhus M, Lund E (2008). Childhood diet in relation to Sami and Norwegian ethnicity in northern and mid-Norway – the SAMINOR study. Public Health Nutr.

[2] Nystad T, Melhus M, Brustad M, Lund E (2010). Ethnic differences in the prevalence of general and central obesity among the Sami and Norwegian populations: the SAMINOR study. Scand J Public Health.

[3] Westlund K (1979). The cardiovascular study in Finnmark 1974–75: report no. 25.

[4] Utsi E, Bonaa K (1998). Koronar hjertesykdom hos samiskættede og norskættede i Finnmark (Coronary heart diseases among Lapps and Norwegians in Finnmark). Tidsskr Nor Laegeforen.

[5] Njølstad I, Arnesen E, Lund-Larsen P (1998). Cardiovascular diseases and diabetes mellitus in different ethnic groups: the Finnmark study. Epidemiology.

[6] Thelle DS, Førde OH, Arnesen E (1982). Distribution of high-density lipoprotein cholesterol according to age, sex, and ethnic origin: cardiovascular disease study in Finnmark 1977. J Epidemiol Community Health.

[7] Thelle DS, Førde OH (1979). The cardiovascular study in Finnmark county: coronary risk factors and the occurrence of myocardial infarction in first degree relatives and in subjects of different ethnic origin. Am J Epidemiol.

[8] Førde O, Thelle D, Miller N, Mjøs O (1978). The Tromsø heart study. Distribution of serum cholesterol between high density and lower density lipoproteins in subjects of Norse, Finnish and Lappish ethnic origin. Acta Medica Scand.

[9] Nystad T (2010). A population-based study on cardiovascular risk factors and self-reported type 2 diabetes mellitus in the Sami population.

[10] Nystad T, Utsi E, Selmer R, Brox J, Melhus M, Lund E (2008). Distribution of apoB/apoA-1 ratio and blood lipids in Sami, Kven and Norwegian populations: the SAMINOR study. Int J Circumpolar Health.

[11] Tynes T, Haldorsen T (2007). Mortality in the Sami population of North Norway, 1970–98. Scand J Public Health.

[12] Tverdal A (1997). Cohort study of ethnic group and cardiovascular and total mortality over 15 years. J Clin Epidemiol.

[13] Lund E, Melhus M, Hansen K, Nystad T, Broderstad AR, Selmer R (2007). Population based study of health and living conditions in areas with both Sami and Norwegian populations – the SAMINOR study. Int J Circumpolar Health.

[14] Niemi E, Tägil S (1995). The Finns in Northern Scandinavia and minority policy. Ethnicity and nation building in the Nordic world.

[15] Eliassen B-M (2013). Social determinants of self-rated health and cardiovascular disease among the Sami and other Arctic indigenous peoples: the SLiCA study and the SAMINOR study.

[16] WHO (1999). Definition, diagnosis and classification of diabetes mellitus and its complications. Part 1: diagnosis and classification of diabetes mellitus.

[17] Eurostat (2013). Revision of the European standard population – report of Eurostat's task force.

[18] Mannan H, Stevenson C, Peeters A, Walls H, McNeil J (2010). Framingham risk prediction equations for incidence of cardiovascular disease using detailed measures for smoking. Heart Int.

[19] Edin-Liljegren A, Hassler S, Sjölander P, Daerga L (2004). Risk factors for cardiovascular diseases among Swedish Sami – a controlled cohort study. Int J Circumpolar Health.

[20] Ross AB, Johansson A, Vavruch-Nilsson V, Hassler S, Sjolander P, Edin-Liljegren A (2009). Adherence to a traditional lifestyle affects food and nutrient intake among modern Swedish Sami. Int J Circumpolar Health.

[21] Hassler S, Johansson R, Sjölander P, Grönberg H, Damber L (2005). Causes of death in the Sami population of Sweden, 1961–2000. Int J Epidemiol.

[22] Sjölander P, Hassler S, Janlert U (2008). Stroke and acute myocardial infarction in the Swedish Sami population: incidence and mortality in relation to income and level of education. Scand J Public Health.

[23] Jørgensen M, Bjerregaard P, Kjaergaard J, Borch-Johnsen K (2008). High prevalence of markers of coronary heart disease among Greenland Inuit. Atherosclerosis.

[24] Eliassen BM, Graff-Iversen S, Melhus M, Lochen ML, Broderstad AR (2014). Ethnic difference in the prevalence of angina pectoris in Sami and non-Sami populations: the SAMINOR study. Int J Circumpolar Health.

[25] Machon M, Arriola L, Larranaga N, Amiano P, Moreno-Iribas C, Agudo A (2013). Validity of self-reported prevalent cases of stroke and acute myocardial infarction in the Spanish cohort of the EPIC study. J Epidemiol Community Health.

[26] Njølstad I (1998). Incidence of and risk factors for myocardial infarction, stroke, and diabetes mellitus in a general population: the Finnmark study 1974–1989.

[27] Tretli S, Lund-Larsen P, Foss O (1982). Reliability of questionnaire information on cardiovascular disease and diabetes: cardiovascular disease study in Finnmark county. J Epidemiol Community Health.

[28] Ingstad B (2007). Medisinsk antropologi: en innføring.

[29] Converse JM, Presser S (1986). Survey questions/handcrafting the standardized questionnaire.

